# Generation-Specific Heterosis in Lactation, Reproduction, and Blood Transcriptomic Profiles of Chinese Simmental × Holstein Crossbred Cows

**DOI:** 10.3390/ani16121892

**Published:** 2026-06-18

**Authors:** Hongkun Zhao, Jingjing Wen, Jiajie Huang, Xiaoyun Liang, Qiuming Chen, Lei Xu

**Affiliations:** College of Animal Science, Xinjiang Agricultural University, Urumqi 830052, China; zhaohongkun4416@163.com (H.Z.); w18293472033@126.com (J.W.); jayjayh@yeah.net (J.H.); 15823168822@163.com (X.L.); cqm19860612@126.com (Q.C.)

**Keywords:** Chinese Simmental, Chinese Holstein, crossbreeding, heterosis, lactation performance, reproductive performance, hematological traits, blood transcriptome, non-additive expression

## Abstract

This study investigated generation-specific heterosis in Chinese Simmental × Chinese Holstein crossbred cows by comparing lactation performance, reproductive performance, hematological traits, and blood transcriptomic profiles among Chinese Simmental (SIM), Chinese Holstein (HOL), F1 crossbreds, and first-generation backcrosses (BC1). Lactation analysis was based on 17,005 valid records, reproductive analysis was based on 5481 valid records, and transcriptomic analysis was conducted using blood samples from 31 cows. F1 showed the most favorable reproductive profile, whereas BC1 showed relatively strong milk-yield performance. Hematological analysis revealed group-level differences in leukocyte-related indicators. Main-text transcriptomic interpretation focused on SIM, F1, and BC1 comparisons because the HOL RNA-seq group included only three samples. These comparisons suggested that F1-related DEGs were mainly associated with immune and inflammatory processes, whereas BC1-related DEGs showed broader immune- and metabolism-related enrichment patterns. Candidate-gene expression heterosis and d/a-ratio analyses are presented as exploratory supplementary results with bootstrap uncertainty estimates because qPCR validation was not available. Overall, these results indicate that heterosis in this crossbreeding system is generation-specific rather than uniform. The integration of phenotypic and blood transcriptomic analyses provides candidate pathways and associated systemic expression patterns for future validation rather than definitive mechanistic evidence.

## Simple Summary

Crossbreeding is an important strategy for improving production performance, reproductive efficiency, and environmental adaptability in cattle, but different crossbred generations may not show the same advantages. In this study, Chinese Simmental, Chinese Holstein, first-generation crossbreds, and first-generation backcross cows raised under the same production conditions at Hutubi Cattle Breeding Farm in Xinjiang were compared in terms of lactation performance, reproductive performance, hematological traits, and blood transcriptomic profiles. The results showed that first-generation crossbreds exhibited clearer advantages in reproductive performance, whereas first-generation backcross cows showed better overall lactation balance. Blood transcriptomic analysis suggested that expression differences associated with first-generation crossbreds were mainly related to immune and inflammatory processes, whereas first-generation backcross cows showed broader enrichment involving immune- and metabolism-related pathways. These findings indicate that heterosis patterns in this crossbreeding system are generation-specific and should be interpreted as exploratory transcriptomic associations.

## 1. Introduction

Intensive selection of dairy cattle for milk production has substantially increased milk output over recent decades; however, this progress has often been accompanied by unfavorable correlated responses in fertility, health, and longevity [1,2]. Crossbreeding has therefore regained interest as a strategy to combine the production potential of specialized dairy breeds with the functional robustness of other breeds [3]. In dairy systems, crossbreeding is particularly valued for heterosis and breed complementarity, especially for low-heritability traits such as fertility, survival, and health, for which non-additive genetic effects may be more evident than for production traits [4].

Chinese Holstein is the dominant specialized dairy breed in China and is widely used for high milk production, whereas Chinese Simmental is an important dual-purpose breed with favorable adaptability and functional performance [5,6]. From a breeding perspective, combining these two genetic backgrounds may help improve the balance between milk yield and functional traits. Previous studies in dairy cattle have shown that crossbred cows often outperform pure Holsteins in fertility and survival, even when milk production remains intermediate or closer to the Holstein type [7,8]. Studies involving Simmental or other dual-purpose breeds have also suggested that these genetic backgrounds can contribute favorable reproductive and functional characteristics in crossbreeding systems [9,10]. However, different crossbred generations may show complementary rather than uniform advantages, with differences among pure Holstein, F1, and later-generation crossbreds in milk production, fertility, and udder-health traits [11]. These findings indicate that heterosis in dairy crossbreeding is both trait-dependent and generation-dependent.

Although phenotypic heterosis has been widely documented in dairy cattle, its molecular basis remains incompletely understood. Blood is increasingly recognized as a useful tissue for systemic transcriptomic analysis because it can reflect immune, metabolic, and physiological status at the whole-animal level [12]. In cattle, blood transcriptome studies have revealed breed- and performance-related differences in immune and metabolic pathways [13,14,15]. For example, whole-blood transcriptome profiling has identified distinct expression patterns between Italian Holstein and Italian Simmental cows [13], between cattle breeds differing in adaptive response [14], and between Chinese Holstein cows with divergent milk yield [15]. These studies support the use of blood transcriptomic data to explore breed-associated expression patterns and candidate pathways related to economically important traits.

In crossbred populations, transcriptomic analysis also provides an opportunity to describe expression heterosis and non-additive expression patterns. Hybrid gene expression has been described using additive, dominance-like, and overdominance-like categories [16]. However, such classifications depend on sampling precision and therefore require cautious interpretation in small RNA-seq cohorts. Integrating phenotypic heterosis with blood transcriptomic analysis may help characterize how different crossbred generations differ not only in performance but also in associated systemic expression patterns and functional pathways.

Based on this background, the present study compared Chinese Simmental, Chinese Holstein, F1, and BC1 cows with respect to lactation performance, reproductive performance, hematological traits, and blood transcriptomic profiles. The objectives were to: (i) evaluate generation-specific heterosis in lactation and reproductive traits; (ii) characterize differences in hematological indicators and blood transcriptomic profiles among genetic groups; (iii) identify major GO and KEGG enrichment patterns associated with crossbred generations; and (iv) describe exploratory expression heterosis and genetic-effect classifications of selected candidate genes.

This study integrates phenotypic and blood transcriptomic data to explore molecular patterns associated with generation-specific heterosis. Because peripheral blood transcriptomes primarily reflect systemic physiological status, the transcriptomic results are interpreted as whole-animal-level associations rather than tissue-specific regulatory mechanisms.

## 2. Materials and Methods

### 2.1. Animals, Herd Conditions, Data Sources, and Sampling

This study was conducted at Hutubi Cattle Breeding Farm in Hutubi County, Xinjiang, China. Four genetic groups were included: Chinese Simmental (SIM), Chinese Holstein (HOL), first-generation crossbred cows (F1), and first-generation backcross cows (BC1). F1 cows were generated by mating Chinese Simmental sires with Chinese Holstein dams, whereas BC1 cows were generated by mating F1 females with Chinese Simmental sires. SIM and HOL cows were included as parental reference populations and were also analyzed as experimental groups under the same management conditions to enable direct phenotypic and transcriptomic comparisons across generations.

All animals were maintained under the same commercial production conditions. Cows were housed in a loose-housing system, fed a uniform total mixed ration, had free access to water, and were managed according to the routine health and immunization program of the farm. The farm is located in a temperate continental arid to semi-arid region.

Phenotypic analyses were based on farm production records and blood-based sampling. Lactation trait analysis used production records collected from 2022 to 2024, whereas reproductive trait analysis used records collected from 2017 to 2025. After quality control, 17,005 lactation records and 5481 reproductive records were retained for analysis.

Hematological and transcriptomic analyses were performed using blood samples collected from 31 clinically healthy lactating cows, including 10 SIM, 3 HOL, 10 F1, and 8 BC1 individuals. All sampled cows were maintained under the same herd conditions and sampled within the same management period. The sampled animals were clinically healthy, in mid-lactation, and free from evident metabolic or reproductive disorders. Individuals were randomly selected within each genetic group while controlling for parity and lactation stage as far as possible to reduce potential confounding effects.

Because this herd was developed primarily under a Simmental-based crossbreeding system, the number of available purebred Holstein cows for transcriptomic sampling was limited, resulting in a relatively small HOL subgroup. This limited sample size (*n* = 3) may reduce the statistical power for detecting differentially expressed genes (DEGs), particularly in comparisons involving HOL, and may lead to unstable estimates and false-negative findings. Therefore, HOL-related DEG comparisons were not used for main biological inference and were reported only as exploratory supplementary results.

### 2.2. Phenotypic Trait Definition and Statistical Analysis

The phenotypic traits analyzed in this study included lactation and reproductive traits. Lactation traits comprised 305-day milk yield (305MY), milk fat percentage (MFP), milk protein percentage (MPP), somatic cell count (SCC), and milk urea nitrogen (MUN). Reproductive traits included first-service conception rate (FSCR), overall conception rate (OCR), services per conception (SPC), days open (DO), calving interval (CI), gestation length (GL), and age at first calving (AFC).

Raw records were screened by removing missing values, duplicated records, outliers, and biologically unreasonable observations according to predefined quality-control criteria (Table 1). Records with fewer than 305 days in milk were adjusted to 305MY using the farm’s standard correction procedure. SCC was retained in its original biological unit (×10^4^ cells/mL) to facilitate biological interpretation and heterosis calculation, and because SCC is widely used as an indicator of udder health and milk quality in dairy cattle [17].

Continuous lactation and reproductive traits were analyzed using mixed models to account for the record structure, following commonly used approaches for evaluating production and reproductive parameters in dairy cows [18]. For continuous traits with repeated records, animal identity was included as a random effect to account for repeated observations from the same cow. Genetic group, calving year, calving season, and parity were fitted as fixed effects where applicable. For binary reproductive traits, including FSCR and OCR, generalized linear models with a binomial distribution and logit link were used, with the same fixed effects where applicable. AFC was analyzed separately because it is recorded once per animal.

For continuous traits other than AFC, the following mixed model was fitted:

$$Y_{ijklm}=\mu +G_{i}+Y_{j}+S_{k}+A_{m}+P_{l}+e_{ijklm}$$

where $Y_{ijklm}$ is the observed continuous trait, μ is the overall mean, $G_{i}$ is the fixed effect of genetic group, $Y_{j}$ is the fixed effect of calving year, $S_{k}\;$is the fixed effect of calving season, $P_{l}$ is the fixed effect of parity, $A_{m}$ is the random animal effect, and $e_{ijklm}$ is the residual error. The random animal effect was included to account for repeated records from the same cow.

For AFC, which is recorded only once per animal, a reduced model was used that included genetic group, year of first calving, and season of first calving as fixed effects.

All statistical analyses of phenotypic traits were performed in R (version 4.2.5). The main packages used included lme4 (v1.1-34) for mixed models, emmeans (v1.8.9) for least-squares mean estimation, and ggplot2 (v3.4.4) for visualization. Differences were considered significant at *p* < 0.05.

### 2.3. Heterosis Calculation

Heterosis in the F1 group was evaluated using mid-parent heterosis, in which the mean value of the two parental breeds was used as the reference to assess the deviation of F1 performance from the parental average, following standard formulations used in heterosis studies [19]. The mid-parent value (MPV) was calculated as:

$$MPV=\frac{SIM+HOL}{2}$$

The heterosis rate of F1 was then calculated as:

$$H_{\bar{F1}}\left(\%\right)=\frac{\left(F1-MPV\right)}{\left(MPV\right)}\times 100$$

where $H_{\bar{F1}}$ is the heterosis rate of the F1 group, F1 is the mean value of the corresponding trait in F1 cows, and SIM and HOL are the mean values of the corresponding trait in the Chinese Simmental and Chinese Holstein parental groups, respectively. Positive values indicate that the F1 mean exceeded the parental average, whereas negative values indicate that the F1 mean was lower than the parental average.

Because BC1 represented a first backcross generation derived from mating F1 females with Chinese Simmental sires, its expected breed composition was 75% Chinese Simmental and 25% Chinese Holstein. Therefore, instead of applying the same mid-parent formula used for F1, the expected additive value of BC1 was defined according to the breed proportion [2,20]

$${EP}_{BC1}=0.75\times SIM+0.25\times HOL$$

The heterosis rate of BC1 was calculated as:

$$H_{\bar{BC1}}\left(\%\right)=\frac{\left(BC1-{EP}_{BC1}\right)}{{EP}_{BC1}}\times 100$$

where $H_{\bar{BC1}}$ is the heterosis rate of the BC1 group, BC1 is the mean value of the corresponding trait in BC1 cows, and ${EP}_{BC1}$ is the expected additive value of the backcross generation. This formulation reflects the fact that BC1 is a backcross rather than a direct first-generation cross and is consistent with breed-proportion-based heterosis models used in dairy cattle studies [2,20].

In this study, heterosis rates were treated as descriptive indices. For lactation traits, approximate standard errors for heterosis percentages were derived by the delta method from the least-squares means and their standard errors, and these uncertainty estimates are presented together with the lactation heterosis results. Statistical significance was inferred from the model-based comparisons among genetic groups described above.

### 2.4. RNA Extraction, Sequencing, and Expression Quantification

Peripheral blood samples (10 mL) were collected into EDTA anticoagulant tubes. RNA extraction and library construction were performed by Novogene (Beijing, China), and sequencing was conducted on the Illumina PE150 platform.

Raw sequencing reads were subjected to quality assessment using FastQC (v0.11.9) [21]. Adapter sequences, reads containing excessive unknown bases (N), and low-quality reads were removed to obtain clean reads. Reference genome indices were constructed based on the bovine reference genome ARS-UCD1.2. Clean reads were first aligned to the reference genome using STAR (v2.7.10a) [1]. Reads that remained unmapped after STAR alignment were extracted and subjected to a second-round alignment using HISAT2 (v2.2.1) with known splice-site information to improve the overall mapping rate [22].

The SAM files generated by STAR and HISAT2 were processed using Picard tools, including CleanSam and MergeSamFiles [23]. Based on the merged BAM files, transcript assembly and expression quantification were performed using StringTie (v2.2.1) [24], and gene- and transcript-level expression matrices were generated for downstream analyses.

As an additional transcriptomic quality-control and sample-structure assessment, the FPKM matrix was transformed as log2(FPKM + 1), and genes expressed at FPKM > 1 in at least three samples were retained. The 5000 most variable retained genes were used for principal component analysis (PCA) and exploratory k-means clustering (k = 3). Old sample labels inferred from sample-name prefixes were used only as reference information rather than as definitive relabeling criteria, because a small number of old labels could be inaccurate and the HOL group contained only three samples.

### 2.5. Differential Expression, Functional Enrichment, and Candidate Gene Analysis

Pairwise differential expression analysis was initially performed among the four genetic groups. However, because the HOL RNA-seq subgroup contained only three samples, DEG comparisons involving HOL were not used for main-text biological inference. The main DEG and enrichment interpretation focused on SIM vs. F1 and SIM vs. BC1. The F1 vs. BC1 comparison, which yielded only a small DEG set, was treated as an exploratory supplementary result together with HOL-related comparisons. HOL-related DEG results were moved to the Supplementary Materials and are presented only as exploratory descriptive outputs. Gene- and transcript-level abundance estimates generated by StringTie were imported into the ballgown package for downstream analysis [25]. Differential expression analysis was performed in R based on the expression matrix, and genes with a Benjamini–Hochberg adjusted *p* value < 0.05 and |log2 fold change| > 1 were considered differentially expressed.

Functional enrichment analysis was conducted for comparisons with sufficient DEG numbers and clear biological relevance. GO and KEGG enrichment analyses were performed using the DAVID database [26]. Multiple testing correction was performed using the Benjamini–Hochberg false discovery rate (FDR) method, and enriched GO terms or KEGG pathways with adjusted *p* < 0.05 were considered significant.

Putative candidate genes were selected from DEGs using predefined criteria: significant differential expression after FDR correction, relatively large expression differences, involvement in immune-, metabolic-, lipid-transport-, or reproduction-related GO/KEGG categories, and biological relevance to lactation, reproduction, or systemic physiological status based on published evidence. Individual-level gene count matrices recovered from the RNA-seq analysis directory were used to estimate uncertainty for expression heterosis and d/a ratios by non-parametric bootstrap resampling within genetic groups. Because qPCR validation was not performed in the present study, these genes are referred to as putative candidate genes, and the expression heterosis and genetic-effect classifications were moved to the Supplementary Materials and interpreted as exploratory analyses.

All transcriptome-related statistical analyses were performed in RStudio (version 4.2.5). Given the limited sample size in some groups, especially HOL, this study focused on identifying major transcriptomic patterns rather than exhaustive DEG detection.

## 3. Results

### 3.1. Lactation Performance Differed Among Genetic Groups

As shown in Table 2, significant differences among genetic groups were observed for 305-day milk yield, milk fat percentage, milk protein percentage, and milk urea nitrogen (*p* < 0.001), whereas somatic cell count did not differ significantly among groups (*p* = 0.120). HOL showed the highest 305-day milk yield, while both crossbred groups remained clearly above SIM. In contrast, SIM showed relatively higher milk fat and milk protein percentages, whereas F1 and BC1 generally displayed intermediate values between the two parental breeds. F1 showed the lowest milk urea nitrogen level among the four groups.

**Figure 1:**
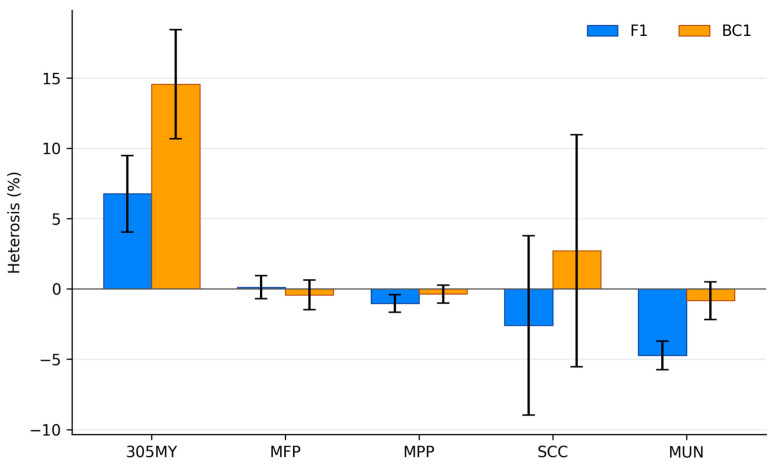
Descriptive heterosis percentages for lactation traits in F1 and BC1 cows. Notes: Bars show descriptive heterosis percentages for major lactation traits. Error bars indicate approximate standard errors calculated by the delta method from the least-squares means and their standard errors in Table 2. F1 heterosis was calculated relative to the mid-parent value, whereas BC1 heterosis was calculated relative to the expected 75% SIM and 25% HOL additive value. Positive values indicate positive heterosis, whereas negative values indicate negative heterosis. For traits in which lower values are biologically favorable, negative heterosis indicates an advantageous change.

As shown in Figure 1, descriptive heterosis analysis further supported these patterns. Both F1 and BC1 showed positive heterosis estimates for 305-day milk yield, reaching 7.5% and 8.6%, respectively, whereas heterosis estimates for milk fat and milk protein percentages were weak or close to zero. In contrast, both crossbred groups showed favorable negative heterosis estimates for milk urea nitrogen, with a larger negative estimate in F1 than in BC1. Overall, crossbreeding had a more evident effect on milk yield than on milk composition traits.

### 3.2. Reproductive Performance Differed Among Genetic Groups

As shown in Table 3, significant differences among genetic groups were observed for overall conception rate (OCR), services per conception (SPC), days open (DO), calving interval (CI), gestation length (GL), and age at first calving (AFC) (*p* ≤ 0.001), whereas first-service conception rate (FSCR) did not differ significantly among groups (*p* = 0.192).

**Figure 2:**
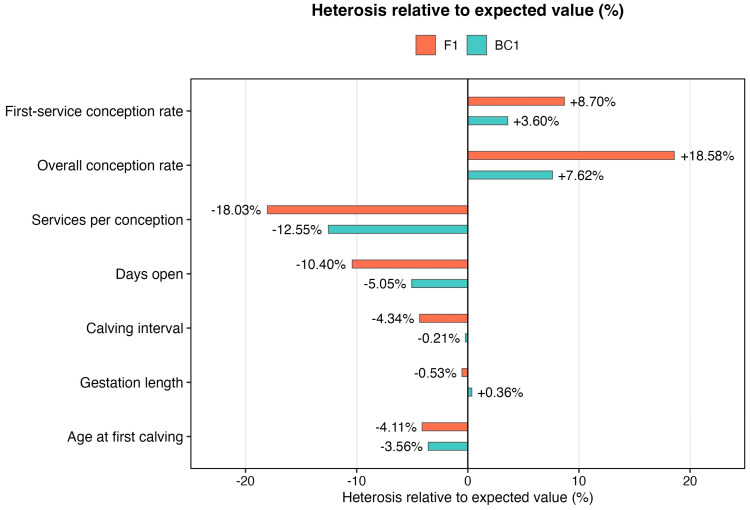
Descriptive heterosis percentages for reproductive traits in F1 and BC1 cows. Notes: Bars show descriptive heterosis percentages for reproductive traits in F1 and BC1 cows. F1 heterosis was calculated relative to the mid-parent value, whereas BC1 heterosis was calculated relative to the expected 75% SIM and 25% HOL additive value. FSCR, first-service conception rate; OCR, overall conception rate; SPC, services per conception; DO, days open; CI, calving interval; GL, gestation length; AFC, age at first calving.

As shown in Figure 2, descriptive heterosis analysis showed F1 showed the highest OCR and the lowest SPC, whereas BC1 showed intermediate values. Similarly, F1 had the shortest DO and CI, while SIM showed the highest values for these interval-related traits. Meanwhile, positive heterosis estimates for conception-related traits and favorable negative heterosis estimates for interval-related traits, including SPC, DO, CI, and AFC. The magnitude of heterosis was generally larger in F1 than in BC1, suggesting that the reproductive advantage was more evident in the first crossbred generation.

### 3.3. Hematological Indicators Varied Among the Four Genetic Groups

As shown in Table 4, significant differences among genetic groups were observed for leukocyte-related traits, including WBC, lymphocyte count, and monocyte count (*p* < 0.05), whereas RBC, HGB, HCT, and PLT did not differ significantly. F1 showed the highest values for WBC, lymphocyte count, and monocyte count, with corresponding heterosis estimates of 92.18%, 102.52%, and 100.00%, respectively. In BC1, these leukocyte-related traits showed smaller positive heterosis estimates, ranging from 24.29% to 33.33%.

For erythrocyte- and platelet-related traits, variation among groups was limited, and no significant differences were detected. Overall, hematological divergence among genetic groups was mainly reflected in leukocyte-related indicators rather than erythrocyte- or platelet-related traits.

### 3.4. RNA-Seq Data Quality Was Sufficient for Downstream Analysis

Exploratory PCA and k-means clustering (k = 3) based on the FPKM matrix were used to assess whether sample-label information was broadly consistent with expression structure (Supplementary Figure S1). The three expression-defined clusters should not be interpreted as three pure breeds. The largest SIM-like cluster contained all ten A-labeled samples and therefore most likely corresponded to the Chinese Simmental expression background. A second cluster contained mainly C- and D-labeled samples, especially D/BC1 samples, and was interpreted as a crossbred-like group closer to BC1. The remaining cluster consisted of C2 alone; because C2 showed the closest expression similarity to the F1 centroid, it was treated as an F1-like outlier rather than as a separate breed. The three HOL-labeled samples did not form a stable independent cluster, which is consistent with the small HOL RNA-seq sample size and supports cautious interpretation of HOL-related transcriptomic comparisons.

### 3.5. Differentially Expressed Genes Showed Distinct Divergence Patterns Among Non-HOL Groups

To avoid overinterpretation caused by the limited HOL RNA-seq sample size, the main-text DEG analysis focused only on comparisons among SIM, F1, and BC1. HOL-related DEG outputs are provided in Supplementary Table S5 and Supplementary Figure S2 as exploratory descriptive results and were not used for main biological inference.

**Figure 3:**
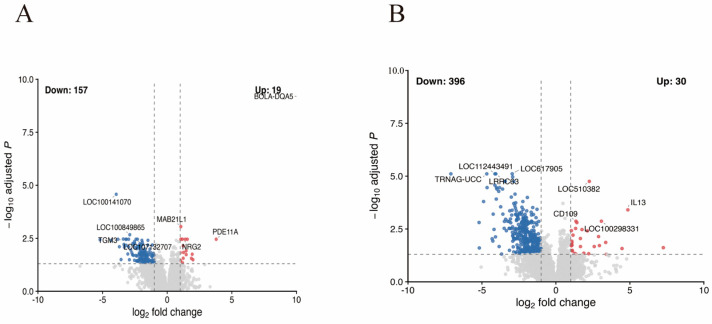
Volcano plots of differentially expressed genes for the two main non-HOL comparisons. Notes: Volcano plots are shown for the two main non-HOL comparisons: (**A**) SIM vs. F1 and (**B**) SIM vs. BC1. The F1 vs. BC1 comparison identified only a small DEG set and was provided in the Supplementary Materials as an exploratory descriptive result. Comparisons involving HOL were moved to Supplementary Figure S2 and Supplementary Table S5 and should be interpreted as exploratory descriptive results because the HOL RNA-seq group contained only three samples.

The corresponding volcano plots are shown in Figure 3. Among the main-text comparisons, SIM vs. F1 showed 175 DEGs, including 157 downregulated and 18 upregulated genes. SIM vs. BC1 showed 137 DEGs, including 109 downregulated and 28 upregulated genes. These results indicate that F1 and BC1 differed in their blood-expression divergence relative to SIM.

### 3.6. Functional Enrichment Patterns of Differentially Expressed Genes Among Genetic Groups

**Figure 4:**
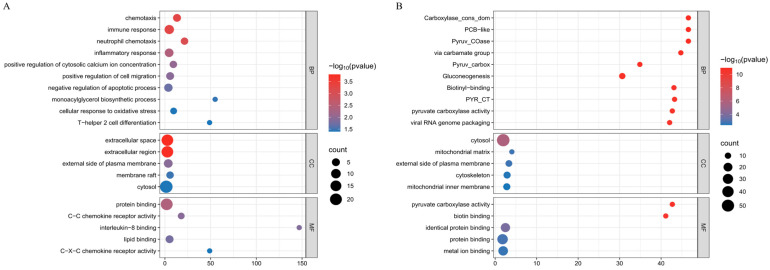
GO enrichment analysis of differentially expressed genes for the two main non-HOL comparisons. Notes: (**A**) GO enrichment results for DEGs identified between SIM and F1. (**B**) GO enrichment results for DEGs identified between SIM and BC1. The *x*-axis indicates the number of enriched genes, and the *y*-axis indicates GO term names. Bubble size represents gene count, and bubble color indicates enrichment significance as −log10 (*p* value). BP, biological process; CC, cellular component; MF, molecular function.

GO enrichment analysis suggested distinct functional patterns between the SIM vs. F1 and SIM vs. BC1 comparisons (Figure 4). In SIM vs. F1, enriched GO terms were mainly associated with immune- and inflammation-related functions, including chemotaxis, immune response, and neutrophil chemotaxis. In contrast, SIM vs. BC1 showed enrichment in metabolism-related terms, including pyruvate carboxylase-related processes, gluconeogenesis, and biotin binding.

**Figure 5:**
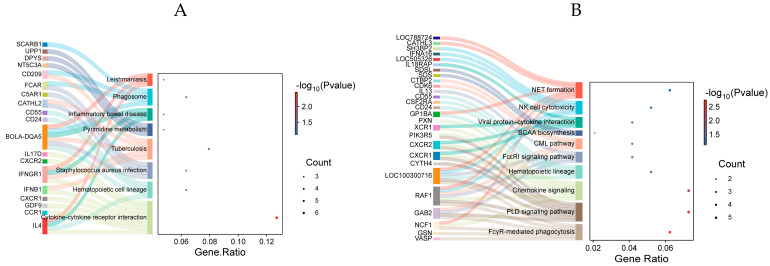
KEGG enrichment analysis of differentially expressed genes for the two main non-HOL comparisons. Notes: KEGG enrichment results are shown for (**A**) SIM vs. F1 and (**B**) SIM vs. BC1. In each panel, the left plot shows gene–pathway relationships, and the right bubble plot shows KEGG pathway enrichment. The x-axis indicates the rich factor, bubble size represents gene count, and bubble color indicates enrichment significance as −log10(p value). Additional enrichment outputs involving HOL or the small F1 vs. BC1 DEG set were moved to Supplementary Figure S3 and should be interpreted as exploratory descriptive information. Adjusted *p* values were calculated using Benjamini–Hochberg FDR correction.

KEGG enrichment analysis suggested comparison-specific pathway patterns for the two main non-HOL comparisons (Figure 5). In SIM vs. F1, enriched pathways were mainly related to immune and infection-associated functions, including cytokine–cytokine receptor interaction, hematopoietic cell lineage, phagosome, and tuberculosis. In SIM vs. BC1, a broader set of pathways was enriched, involving immune-related pathways such as chemokine signaling and natural killer cell-mediated cytotoxicity, as well as metabolism-related pathways such as branched-chain amino acid biosynthesis and cAMP signaling. Comparisons with smaller DEG sets showed fewer enriched pathways and were interpreted descriptively. Additional enrichment outputs for HOL-related or very small DEG comparisons are provided in Supplementary Figure S3 and are descriptive only.

### 3.7. Putative Candidate Genes Showed Exploratory Expression Heterosis Patterns

Exploratory expression heterosis was evaluated for 10 putative candidate genes selected using the predefined DEG, pathway-membership, fold-change, and biological-relevance criteria described in the Methods. In response to the limited RNA-seq sample size and the absence of qPCR validation, the expression heterosis values and d/a-ratio classifications were moved to Supplementary Tables S1 and S2. Bootstrap standard errors and 95% confidence intervals were added to these supplementary tables. Because several confidence intervals were wide, these results are presented as preliminary observations requiring validation in larger populations and independent expression assays.

## 4. Discussion

Crossbreeding in dairy cattle is generally used to exploit heterosis and breed complementarity, particularly when breeding objectives extend beyond milk yield to include fertility, survival, and overall functional efficiency [27,28]. In the present study, the four genetic groups showed distinct phenotypic profiles and associated blood transcriptomic patterns. HOL maintained the highest lactation level, F1 showed the most favorable reproductive profile, and BC1 exhibited a lactation pattern closer to HOL while retaining part of the crossbred advantage. These findings suggest that heterosis in this Chinese Simmental × Holstein crossbreeding system was generation-specific rather than uniform. This pattern agrees with previous dairy crossbreeding studies showing that crossbreeding is especially valuable when functional traits are evaluated together with production traits rather than in isolation [27,28].

The lactation results suggested that the backcross generation retained a more Holstein-like production pattern. BC1 was closer to HOL than F1 in overall lactation performance, whereas F1 remained more intermediate. Similar trends have been reported in commercial and experimental crossbreeding systems. Favorable heterosis for milk yield has been observed in F1 cows, whereas recombination effects in later-generation or backcross animals may reduce or modify the original heterotic response [16]. In addition, both heterosis and breed proportion have been shown to contribute to milk production and udder-health traits, and later-generation crossbreds may combine functional benefits with production profiles closer to Holstein [11,29]. These reports are consistent with the present results.

By contrast, the reproductive results showed a clearer advantage for the first crossbred generation. F1 displayed the most favorable profile for OCR, SPC, DO, and CI, whereas BC1 retained only part of this advantage. Similar patterns have been documented in previous dairy crossbreeding studies. Crossbred cows have been reported to show higher first-service conception rates and fewer days open than pure Holsteins [30]. A reproductive advantage of crossbreds over Holsteins has also been described for first-service conception rate and survival to second calving [31]. More recently, improvements in first-service conception rate, overall conception rate, and days open have again been reported in crossbred dairy cows compared with pure Holsteins [32]. These findings support the interpretation that reproductive heterosis was more evident in F1 than in BC1 in the present population.

Together, these studies are consistent with the possibility that F1 displayed stronger heterosis-related reproductive patterns, whereas BC1 retained this pattern only partially. The Simmental background of this population is also relevant for interpreting the reproductive contrast between F1 and BC1. In the present study, F1 showed more favorable values than the parental groups for several reproductive traits, whereas BC1 shifted toward a more intermediate pattern. This agrees with evidence that dual-purpose breeds such as Simmental can contribute favorable reproductive performance when crossed with highly specialized dairy breeds. Better fertility intervals have been reported in dual-purpose Simmental cows than in specialized Holsteins [33]. Holstein × Simmental cows have also been shown to have shorter calving intervals, higher conception rates, and shorter calving-to-first-service intervals than pure Holsteins [34]. Moreover, large-scale commercial herd data indicate that crossbred cows can combine acceptable production with improved fertility [35].

The hematological results were consistent with group-level differences in blood-based physiological indicators. Divergence among genetic groups was concentrated mainly in leukocyte-related traits, with F1 showing higher WBC, lymphocyte, and monocyte-related parameters, whereas erythrocyte- and platelet-related traits varied less among groups. Comparable breed-dependent immune-cell differences have previously been reported in cattle blood. Breed-related differences in blood metabolites and immune-cell populations have been observed under heat-stress conditions [36], and breed-specific variation in blood immune-cell composition and cytokine expression has also been described in cattle [37]. At the transcriptomic level, peripheral blood cell analyses in different cattle breeds identified marked differences in gene expression between adaptive-response groups [38]. Additional studies have further shown dynamic changes in blood immune-cell composition under heat stress and transcriptomic differences between purebred cattle and their crossbreds [39,40]. These observations support the interpretation that blood-based indicators can reflect breed- or generation-associated immune and physiological differences, although they should not be interpreted as direct evidence of tissue-specific regulation.

The blood transcriptome results further suggested that F1 and BC1 differed not only in DEG number but also in functional enrichment patterns. The SIM vs. F1 comparison was mainly enriched in immune- and inflammation-related functions, whereas SIM vs. BC1 showed broader enrichment involving both immune-related and metabolism-related pathways. This pattern is consistent with previous RNA-seq studies showing that immune and metabolic pathways are among the most responsive functional categories in blood-derived transcriptomes [41,42,43]. Additional cattle studies of immune epigenomic variation and disease-related whole-blood transcriptomes also support the sensitivity of blood-derived omics data to immune and physiological status [44,45]. Therefore, the transcriptomic results should be interpreted as associated blood-expression patterns and exploratory candidate pathways that may be related to phenotypic differences among crossbred generations, rather than as validated causal mechanisms. Comparisons involving HOL were not used for main-text biological inference because the HOL RNA-seq group contained only three samples.

The putative candidate-gene analysis suggested possible non-additive expression patterns in the crossbred groups, particularly in F1, which is consistent with evidence that dominance and other non-additive effects are common in mammalian gene expression [46]. Moving the expression heterosis and d/a-ratio analyses to the Supplementary Materials, together with bootstrap uncertainty estimates, provides a more cautious presentation of these descriptive indices. However, the wide intervals for several genes and the lack of qPCR validation mean that these findings should not be considered definitive evidence of causal regulation. Instead, the putative candidate genes provide exploratory signals and hypotheses for future validation in larger RNA-seq cohorts and independent expression assays.

Several limitations should be acknowledged. First, transcriptomic analysis was performed using peripheral blood, which reflects systemic immune and metabolic status but does not directly represent gene regulation in target tissues such as the mammary gland, ovary, uterus, or endometrium. Accordingly, the transcriptomic results reflect systemic biological variation rather than tissue-specific regulation. Second, the relatively small HOL sample size reduced the reliability and statistical power of HOL-related DEG analyses. For this reason, HOL-related DEG and enrichment results were moved to the Supplementary Materials and treated as exploratory descriptive information only. Third, although bootstrap-based uncertainty estimates were added for expression heterosis and d/a-ratio analyses, these estimates were derived from a relatively small RNA-seq cohort and several intervals remained wide. Moreover, the candidate-gene results were not independently validated by qPCR or other expression assays. Therefore, these analyses should be interpreted as exploratory and hypothesis-generating rather than confirmatory evidence of causal mechanisms. These limitations indicate that the identified pathways and candidate genes provide a basis for further investigation rather than definitive molecular explanations of heterosis.

## 5. Conclusions

This study systematically compared Chinese Simmental, Chinese Holstein, F1, and BC1 cows in terms of lactation performance, reproductive performance, hematological traits, and blood transcriptomic profiles. The results showed generation-specific differences among the crossbred groups. F1 showed the most favorable reproductive profile, whereas BC1 showed relatively strong milk-yield performance. Hematological differences were mainly reflected in leukocyte-related indicators. Transcriptomic comparisons among SIM, F1, and BC1 suggested generation-associated immune- and metabolism-related expression patterns, whereas HOL-related RNA-seq comparisons were treated as supplementary because of the small HOL sample size.

Overall, these findings indicate generation-specific heterosis patterns in the Chinese Simmental × Chinese Holstein crossbreeding system. The integration of phenotypic and blood transcriptomic data identifies exploratory candidate genes and pathways that require validation in larger cohorts and independent assays. The transcriptomic findings, including the putative candidate genes and supplementary expression heterosis analyses, should be considered preliminary and require validation in larger cohorts and independent assays such as qPCR before being interpreted as definitive molecular evidence of heterosis.

## Figures and Tables

**Table 1:** Quality-control criteria used for screening lactation and reproductive records.

Trait	SIM/F1/BC1	HOL
305MY (kg)	2000~12,000	2000~25,000
MFP (%)	2~7	1.5~9
MPP (%)	2~5	1.5~6
SCC (×10^4^ cells/mL)	0~600	0~600
MUN (mg/dL)	10~18	8~20
AFC (d)	500–1100	510–1080
GL (d)	210–350	240–330
CI (d)	280–600	280–640
DO (d)	30–305	30–305

Notes: 305MY, 305-day milk yield; MFP, milk fat percentage; MPP, milk protein percentage; SCC, somatic cell count; MUN, milk urea nitrogen; AFC, age at first calving; GL, gestation length; CI, calving interval; DO, days open. F1 cows were generated by mating Chinese Simmental sires with Chinese Holstein dams, and BC1 cows were generated by mating F1 females with Chinese Simmental sires.

**Table 2:** Comparison of least-squares means for lactation traits among different genetic groups of dairy cows.

Trait	SIM (9377)	HOL (6651)	F1 (627)	BC1 (324)	*p*-Value
	LSM ± SE	LSM ± SE	LSM ± SE	LSM ± SE	
305MY (kg)	6578.19 ± 85.41 ^a^	8707.20 ± 87.26 ^b^	8159.96 ± 197.77 ^c^	8145.50 ± 265.13 ^bc^	<0.001
MFP (%)	4.11 ± 0.02 ^a^	4.06 ± 0.02 ^b^	4.09 ± 0.03 ^ab^	4.08 ± 0.04 ^ab^	<0.001
MPP (%)	3.42 ± 0.01 ^a^	3.39 ± 0.01 ^b^	3.37 ± 0.02 ^b^	3.40 ± 0.02 ^ab^	<0.001
SCC (×10^4^ cells/mL)	16.67 ± 0.51	17.52 ± 0.52	16.65 ± 1.03	17.34 ± 1.33	0.120
MUN (mg/dL)	15.30 ± 0.10 ^a^	15.29 ± 0.10 ^a^	14.57 ± 0.14 ^b^	15.17 ± 0.19 ^a^	<0.001

Notes: Values are presented as least-squares means (LSM) and standard errors (SE). Different lowercase superscript letters within the same row indicate significant differences among genetic groups (*p* < 0.05). Means sharing at least one common letter do not differ significantly.

**Table 3:** Analysis of differences in reproductive traits among different genetic groups.

Trait	SIM	HOL	F1	BC1	*p*-Value
	LSM ± SE	LSM ± SE	LSM ± SE	LSM ± SE	
FSCR (%)	0.70 ± 0.01	0.68 ± 0.01	0.75 ± 0.03	0.72 ± 0.06	0.192
OCR (%)	0.55 ± 0.01 ^a^	0.58 ± 0.01 ^a^	0.67 ± 0.03 ^b^	0.60 ± 0.05 ^ab^	<0.001
SPC (times)	2.50 ± 0.04 ^b^	2.38 ± 0.05 ^b^	2.00 ± 0.13 ^a^	2.16 ± 0.23 ^ab^	<0.001
DO(d)	144.32 ± 1.82 ^b^	123.34 ± 2.69 ^a^	119.91 ± 8.75 ^a^	132.05 ± 12.23 ^ab^	<0.001
CI (d)	475.99 ± 18.25 ^b^	449.62 ± 18.41 ^a^	442.71 ± 21.13 ^a^	468.39 ± 22.87 ^ab^	<0.001
GL(d)	278.51 ± 0.18 ^a^	279.47 ± 0.26 ^b^	277.52 ± 0.65 ^a^	279.76 ± 1.16 ^ab^	0.001
AFC(d)	756.22 ± 2.58 ^a^	786.88 ± 3.47 ^b^	739.81 ± 6.79 ^a^	736.67 ± 14.80 ^a^	<0.001

Notes: Values are presented as least-squares means (LSM) and standard errors (SE). Different lowercase superscript letters within the same row indicate significant differences among genetic groups (*p* < 0.05). Means sharing at least one common letter do not differ significantly.

**Table 4:** Hematological parameters across genetic groups and heterosis estimates in F1 and BC1 cows.

Trait	SIM	HOL	F1	Heterosis (%)	BC1	Heterosis (%)	*p*-Value
	Mean ± SD	Mean ± SD	Mean ± SD		Mean ± SD		
WBC (10^9^/L)	5.26 ± 0.96 ^a^	4.97 ± 1.67 ^ab^	9.83 ± 1.09 ^b^	92.18	6.61 ± 1.02 ^ab^	29.24	0.023
Lymph (10^9^/L)	1.64 ± 0.35 ^a^	1.53 ± 0.57 ^ab^	3.21 ± 0.37 ^b^	102.52	1.97 ± 0.35 ^ab^	24.29	0.021
Mon (10^9^/L)	0.50 ± 0.09 ^a^	0.40 ± 0.15 ^ab^	0.90 ± 0.10 ^b^	100.00	0.60 ± 0.09 ^ab^	33.33	0.024
RBC (10^12^/L)	5.43 ± 0.45	5.24 ± 0.82	5.85 ± 0.45	9.65	5.61 ± 0.50	5.15	0.886
HGB (g/L)	104.22 ± 4.99	92.33 ± 8.65	101.40 ± 4.74	3.18	94.25 ± 5.30	−4.10	0.451
HCT (%)	28.50 ± 1.42	25.23 ± 2.47	27.86 ± 1.35	3.70	25.67 ± 1.51	−4.44	0.451
PLT (10^9^/L)	342.20 ± 104.88	502.33 ± 191.49	581.90 ± 104.88	37.80	455.12 ± 117.26	7.78	0.461

Notes: Values are presented as mean ± SD. Different lowercase superscript letters within the same row indicate significant differences among genetic groups (*p* < 0.05). Means sharing at least one common letter do not differ significantly. Heterosis (%) was calculated as a descriptive index for F1 and BC1. WBC, white blood cell count; Lymph, lymphocyte count; Mon, monocyte count; RBC, red blood cell count; HGB, hemoglobin; HCT, hematocrit; PLT, platelet count.

## Data Availability

The datasets generated and/or analyzed during the current study are available from the corresponding author (L.X.) upon reasonable request.

## Supplementary Materials

The following supporting information can be downloaded at: https://www.mdpi.com/article/10.3390/ani16121892/s1. Supplementary Figure S1. Exploratory PCA and k = 3 clustering of FPKM-based sample expression profiles. Supplementary Figure S2. Volcano plots of differentially expressed genes among groups with different genetic backgrounds. Supplementary Figure S3. Additional KEGG enrichment outputs for HOL-related or very small DEG comparisons. Supplementary Table S1. Expression heterosis estimates of candidate genes with bootstrap uncertainty. Supplementary Table S2. Genetic-effect classification of candidate genes based on d/a ratios with bootstrap uncertainty. Supplementary Table S3. GO biological process enrichment results for F1 vs. SIM with BH-FDR values. Supplementary Table S4. GO biological process enrichment results for BC1 vs. SIM with BH-FDR values. Supplementary Table S5. HOL-related DEG comparison summary.
