# Supplementary material for: Generation-Specific Heterosis in Lactation, Reproduction, and Blood Transcriptomic Profiles of Chinese Simmental × Holstein Crossbred Cows
Source: Animals (Basel). 2026 Jun 18;16(12):1892. doi: 10.3390/ani16121892 (PMC13295949; doi:10.3390/ani16121892)
Supplement: Supplementary file 1 [file animals-16-01892-s001.zip › animals-4283205-supplementary.pdf]

Supplementary Materials

The supplementary materials are appended at the end of the manuscript because the submission system does not provide a separate upload field for supplementary files. HOL-related transcriptomic results and the detailed expression heterosis/d/a-ratio analyses are provided here as exploratory supplementary materials, mainly because the HOL RNA-seq subgroup contained only three samples and independent qPCR validation was not available.

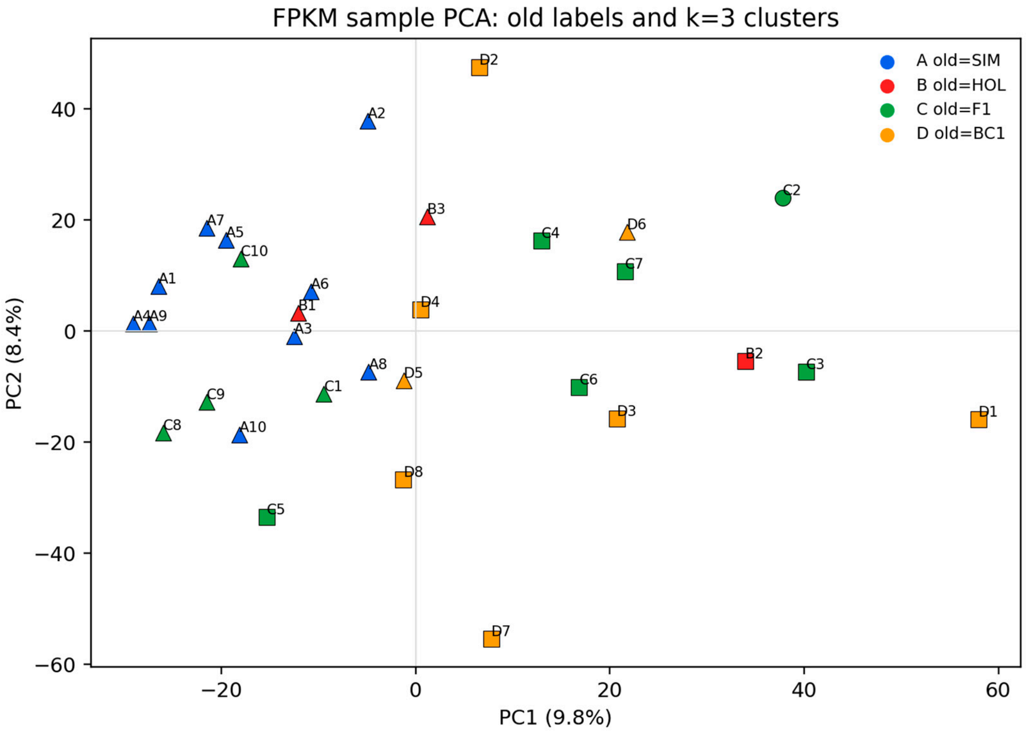

Supplementary Figure S1. Exploratory PCA and k = 3 clustering of FPKM-based sample expression profiles.

Notes: Colors indicate old sample-name-based labels (A = SIM, B = HOL, C = F1, D = BC1), and marker shapes indicate exploratory k = 3 clusters. The clustering was used only to assess broad sample structure and possible label consistency.

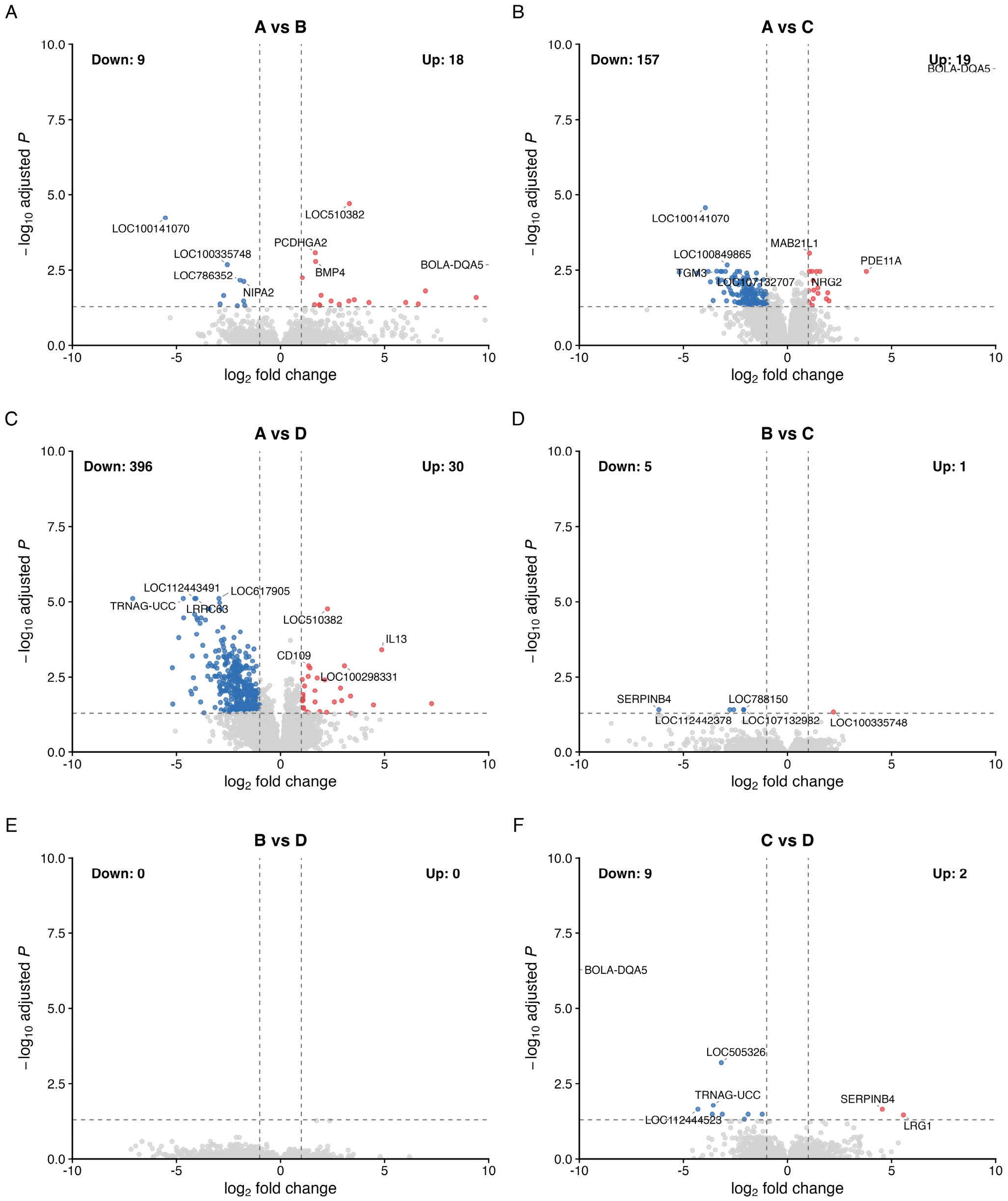

Supplementary Figure S2. Volcano plots of differentially expressed genes among groups with different genetic backgrounds.

Notes: Pairwise comparisons among Chinese Simmental (SIM), Chinese Holstein (HOL), F1, and BC1 are shown for visual reference. Main-text interpretation focuses on SIM vs. F1, SIM vs. BC1, and F1 vs. BC1. Comparisons involving HOL are retained as descriptive visual information only and HOL-related DEG results are summarized in Supplementary Table S5.

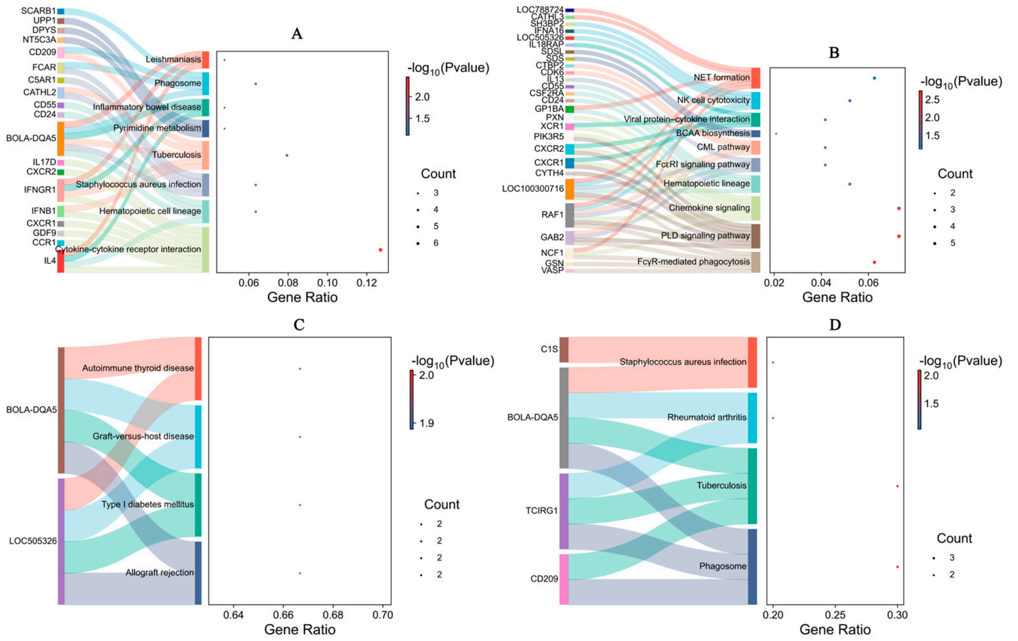

Supplementary Figure S3. KEGG enrichment analysis of differentially expressed genes in selected pairwise comparisons among genetic groups.

Notes: KEGG enrichment results are shown for selected pairwise comparisons. Main-text interpretation emphasizes non-HOL comparisons. HOL-related enrichment outputs, where available, are descriptive only Adjusted p values were calculated using Benjamini-Hochberg FDR correction.

Supplementary Table S1. Expression heterosis estimates of candidate genes with bootstrap uncertainty.

Gene	gene_id	SIM mean	SIM SE	HOL mean	HOL SE	F1 mean	F1 SE	F1 heterosis (%)	F1 heterosis SE	F1 heterosis 95% CI lower	F1 heterosis 95% CI upper	BC1 mean	BC1 SE	BC1 heterosis vs 75SIM25HOL (%)	BC1 heterosis vs 75 SIM 25 HOL SE	BC1 heterosis vs 75 SIM 25 HOL 95% CI lower	BC1 heterosis vs 75 SIM 25 HOL95 % CI upper	BC1 heterosis vs 75 SIM 25 HOL (%)
FABP6	gene10538	12.1	3.25	25.67	3.76	48	14.32	154.19	79.61	24.26	333.54	56	12.82	196.56	73.51	79.92	364.41	261.48
MATN3	gene15144	8.1	1.59	44	15.28	51.4	17.5	97.31	99.19	-27.31	358.82	66.88	14.06	156.72	104.42	39.19	456.24	291.65
DGAT2	gene19310	7144.9	1102.43	16493	7509.5	21443.5	5716.04	81.43	79.44	-14.02	298.92	19123	3346.93	61.8	60.45	-8.25	227.4	101.68
PRR5L	gene19416	74.1	7.44	130.33	64.71	157.9	34.4	54.48	56.27	-18.64	196.47	165.62	33.03	62.03	57.26	-12.13	205.24	87.87
MYBPH	gene19909	50.4	8.69	115.33	58.97	146.2	36.09	76.43	76.74	-15.2	277.13	134.25	19.14	62.01	61.27	-8.12	219.9	101.48
SCARB1	gene21266	4.4	1.02	21.33	15.9	36	14.79	179.79	310.74	-26.67	1140.18	25.75	5.51	100.13	192.3	-17.66	650.06	198.26
CXCR2	gene2232	68.9	10.65	139	35.03	353.5	163.61	240.07	162.67	10.02	630.29	228.62	56.38	119.94	61.02	29.96	264.7	164.54
CXCR1	gene2233	391.9	70.77	929	489.14	1258.1	371.18	90.49	94.57	-16.78	349.81	1068.88	198.12	61.84	67.89	-14.2	241.89	103.14
SLC22A14	gene26966	26.4	4.78	100.67	59.14	77.6	15.2	22.14	70.32	-41.05	220.02	71.75	19.84	12.93	69.32	-52.78	209.95	59.56

Gene	gene_id	SIM mean	SIM SE	HOL mean	HOL SE	F1 mean	F1 SE	F1 heterosis (%)	F1 heterosis SE	F1 heterosis 95% CI lower	F1 heterosis 95% CI upper	BC1 mean	BC1 SE	BC1 heterosis vs 75SIM25HOL (%)	BC1 heterosis vs 75 SIM 25 HOL SE	BC1 heterosis vs 75 SIM 25 HOL 95% CI lower	BC1 heterosis vs 75 SIM 25 HOL95 % CI upper	BC1 heterosis vs 75 SIM 25 HOL (%)
TXNDC2	gene29176	12.6	2.18	26.33	7.62	36.2	7.06	85.96	51.53	9.53	212.26	41.38	10.62	112.54	67.35	13.17	275.44	158.06

Note: Bootstrap SE and 95% CI were calculated by resampling individuals within genetic groups. BC1 heterosis was calculated relative to the expected 75% SIM and 25% HOL additive value, consistent with the definition used in the Methods.

Supplementary Table S2. Genetic-effect classification of candidate genes based on d/a ratios with bootstrap uncertainty.

Gene	gene_id	SIM mean	HOL mean	F1 mean	Mid-parent	d/a ratio	d/a ratio SE	d/a ratio 95% CI lower	d/a ratio 95% CI upper	Putative effect type based on point estimate
FABP6	gene10538	12.1	25.67	48	18.88	4.29	32.74	0.63	14.36	Putative overdominance-like
MATN3	gene15144	8.1	44	51.4	26.05	1.41	3.05	-0.34	10.46	Putative overdominance-like
DGAT2	gene19310	7144.9	16493	21443.5	11818.95	2.06	201.7	-22.55	44.96	Putative overdominance-like
PRR5L	gene19416	74.1	130.33	157.9	102.22	1.98	105.69	-74.92	55.83	Putative overdominance-like
MYBPH	gene19909	50.4	115.33	146.2	82.87	1.95	124.16	-55.45	68.49	Putative overdominance-like
SCARB1	gene21266	4.4	21.33	36	12.87	2.73	205.57	-163.64	138.1	Putative overdominance-like
CXCR2	gene2232	68.9	139	353.5	103.95	7.12	31.46	0.25	34.32	Putative overdominance-like
CXCR1	gene2233	391.9	929	1258.1	660.45	2.23	502.09	-53.7	65.18	Putative overdominance-like
SLC22A14	gene26966	26.4	100.67	77.6	63.53	0.38	40.89	-9.56	18.07	Putative dominance-like
TXNDC2	gene29176	12.6	26.33	36.2	19.47	2.44	33.68	-0.69	18.15	Putative overdominance-like

Note: Bootstrap SE and 95% CI were calculated by resampling individuals within genetic groups. Effect-type labels are based on point estimates and should be interpreted cautiously when confidence intervals are wide.

Supplementary Table S3. GO biological process enrichment results for F1 vs SIM with BH-FDR values.

GO.ID	Term	Annotated	Significant	Expected	Fisher	BH_FDR
GO:0006952	defense response	490	12	2.57	8.2e-06	8.4e-05
GO:0006935	chemotaxis	140	7	0.73	8.4e-06	8.4e-05
GO:0042330	taxis	140	7	0.73	8.4e-06	8.4e-05
GO:0006955	immune response	610	13	3.2	1.5e-05	0.000112
GO:0097529	myeloid leukocyte migration	66	5	0.35	2.4e-05	0.00013
GO:0030595	leukocyte chemotaxis	67	5	0.35	2.6e-05	0.00013
GO:0009605	response to external stimulus	484	11	2.54	4e-05	0.000157
GO:0002376	immune system process	775	14	4.07	4.2e-05	0.000157
GO:0042221	response to chemical	693	13	3.64	5.6e-05	0.000187
GO:0050896	response to stimulus	3908	36	20.5	9e-05	0.00025
GO:0050900	leukocyte migration	88	5	0.46	9.6e-05	0.00025
GO:0060326	cell chemotaxis	89	5	0.47	0.0001	0.00025
GO:0071621	granulocyte chemotaxis	53	4	0.28	0.00017	0.000386
GO:0006950	response to stress	1118	16	5.86	0.00018	0.000386
GO:0097530	granulocyte migration	57	4	0.3	0.00022	0.00044
GO:0040011	locomotion	239	7	1.25	0.00025	0.000469
GO:0006954	inflammatory response	171	6	0.9	0.00027	0.000476
GO:0070887	cellular response to chemical stimulus	353	8	1.85	0.0005	0.000833
GO:0072529	pyrimidine-containing compound catabolic...	9	2	0.05	0.00095	0.0015
GO:0034341	response to type II interferon	39	3	0.2	0.0011	0.00165
GO:0050920	regulation of chemotaxis	42	3	0.22	0.00137	0.00192
GO:0140888	interferon-mediated signaling pathway	11	2	0.06	0.00145	0.00192
GO:0051607	defense response to virus	43	3	0.23	0.00147	0.00192
GO:0002275	myeloid cell activation involved in immu...	12	2	0.06	0.00173	0.00199
GO:0042116	macrophage activation	12	2	0.06	0.00173	0.00199
GO:0009615	response to virus	46	3	0.24	0.00179	0.00199
GO:0030593	neutrophil chemotaxis	46	3	0.24	0.00179	0.00199
GO:0071674	mononuclear cell migration	47	3	0.25	0.0019	0.00204
GO:0002440	production of molecular mediator of immu...	48	3	0.25	0.00202	0.00204
GO:0002702	positive regulation of production of mol...	13	2	0.07	0.00204	0.00204

Note: GO enrichment was performed using topGO classic Fisher tests; BH-FDR values were additionally calculated from Fisher p-values for revised reporting.

Supplementary Table S4. GO biological process enrichment results for BC1 vs SIM with BH-FDR values.

GO.ID	Term	Annotated	Significant	Expected	Fisher	BH_FDR
GO:0098542	defense response to other organism	308	13	2.87	6e-06	9e-05
GO:0009605	response to external stimulus	484	16	4.51	1.1e-05	9e-05
GO:0043207	response to external biotic stimulus	337	13	3.14	1.6e-05	9e-05
GO:0051707	response to other organism	337	13	3.14	1.6e-05	9e-05
GO:0009607	response to biotic stimulus	339	13	3.16	1.7e-05	9e-05
GO:0044419	biological process involved in interspec...	341	13	3.18	1.8e-05	9e-05
GO:0051607	defense response to virus	43	5	0.4	4.7e-05	0.000201
GO:0009615	response to virus	46	5	0.43	6.6e-05	0.000247
GO:0140546	defense response to symbiont	255	10	2.38	0.00014	0.000467
GO:0006952	defense response	490	14	4.57	0.00019	0.00057
GO:0042742	defense response to bacterium	102	6	0.95	0.00038	0.00104
GO:0006565	L-serine catabolic process	4	2	0.04	0.00051	0.00109
GO:0006566	threonine metabolic process	4	2	0.04	0.00051	0.00109
GO:0006567	threonine catabolic process	4	2	0.04	0.00051	0.00109

GO.ID	Term	Annotated	Significant	Expected	Fisher	BH_FDR
GO:0050830	defense response to Gram-positive bacter...	42	4	0.39	0.00061	0.00122
GO:0009408	response to heat	19	3	0.18	0.00069	0.00129
GO:0002376	immune system process	775	17	7.23	0.00086	0.00152
GO:0006910	phagocytosis, recognition	6	2	0.06	0.00126	0.00199
GO:0009068	aspartate family amino acid catabolic pr...	6	2	0.06	0.00126	0.00199
GO:0009266	response to temperature stimulus	24	3	0.22	0.00139	0.00209
GO:0006950	response to stress	1118	21	10.43	0.00152	0.00217
GO:0006955	immune response	610	14	5.69	0.00164	0.00224
GO:0050896	response to stimulus	3908	52	36.45	0.00201	0.00262
GO:0009617	response to bacterium	149	6	1.39	0.00271	0.00339
GO:0006563	L-serine metabolic process	9	2	0.08	0.00298	0.00358
GO:0001562	response to protozoan	11	2	0.1	0.00449	0.00499
GO:0042832	defense response to protozoan	11	2	0.1	0.00449	0.00499
GO:0009071	serine family amino acid catabolic proce...	12	2	0.11	0.00536	0.00574
GO:0030593	neutrophil chemotaxis	46	3	0.43	0.00898	0.00921
GO:0050900	leukocyte migration	88	4	0.82	0.00921	0.00921

Note: The group label was corrected to BC1 after verification against the sample metadata.

Supplementary Table S5. HOL-related DEG comparison summary.

Comparison	Significant DEGs	Upregulated in HOL or first-named group	Downregulated in HOL or first-named group	Interpretation
HOL vs SIM	27	9	18	Descriptive only; HOL RNA-seq n=3
HOL vs F1	6	1	5	Descriptive only; HOL RNA-seq n=3
HOL vs BC1	0	0	0	No significant DEGs; not evidence of no biological difference because HOL RNA-seq n=3

Note: HOL-related DEG results are descriptive only because the HOL RNA-seq group contained three samples, limiting statistical power.
